# Reflective structured dialogue as a tool for addressing wicked public health problems

**DOI:** 10.3389/fpubh.2023.1220029

**Published:** 2023-09-25

**Authors:** Cassidy Weaver, Janaya Brown, Lexi Brady, Parker Carlquist, Seth Dotson, M. Dru Faldmo, P. Cougar Hall, Jeffrey Glenn

**Affiliations:** Department of Public Health, Brigham Young University, Provo, UT, United States

**Keywords:** public health, community health, deliberative inquiry, reflective structured dialogue, higher education

## Abstract

**Introduction:**

Attempts to address wicked public health problems can benefit from collaborative approaches to problem-solving, such as dialogue through structured conversations, that engage a wide range of stakeholders in deliberate inquiry to build trust and mutual understanding. This study seeks to assess the effects of participation in Reflective Structured Dialogue (RSD) on university students’ polarization-related attitudes.

**Methods:**

The BYU Campus Conversations project held 27 structured conversations with 139 participants on three divisive public health topics: COVID-19, mental health, and racism. The conversation structure encouraged students to share their personal experiences and learn from others in an environment that promoted vulnerability and confidentiality.

**Results:**

Pre- and post-conversation surveys measured participant outcomes and found that participation in conversations was strongly associated with improved attitudes related to openness, tribal identity, and moral disdain. Over 95% of participants reported that they enjoyed taking part in the conversations and that it helped them better understand the experiences of others.

**Discussion:**

The results of this project indicate similar conversations could be an effective tool in helping build understanding around divisive public health issues in university and community settings.

## 1. Introduction

Political polarization and conflict over social issues are indelible features of American society. Since the beginning of the COVID-19 pandemic in 2020, critical public health-related problems have increasingly become the focus of many of the most heated debates in legislatures, communities, and schools across the country. In addition to disputes directly associated with the pandemic (e.g., masks, vaccines, school closings, etc.), the past 3 years have seen deepening societal rifts related to police violence, mass shootings, abortion, and other public health concerns. These polarizing issues can be characterized as “wicked” problems, i.e., complex social and political problems with dynamic root causes that are difficult to precisely delineate and that lack definitive and objective solutions, especially across different contexts (1). In contrast with typical problems in disciplines such as mathematics and chemistry, the nature of “wicked” problems is likely to be viewed differently by each stakeholder depending on their values, perspectives, and biases (1, 2).

Polarization affects public health in at least two critical ways. First, polarization has been found to directly affect stress and anxiety, which can lead to a variety of poor health outcomes (3, 4). Second, and perhaps most crucially, polarization impedes collective problem-solving and increases social divisiveness—a factor that can be considered a determinant of health—which creates new and exacerbates existing health and social problems (5, 6). Thus, in order to build a foundation of understanding upon which today’s wicked public health problems can be more effectively resolved, the public health community needs evidence-based approaches for promoting dialogue and reducing polarization around controversial issues.

As opposed to the much more common adversarial or expert-centric approaches typically relied upon by public decisionmakers, addressing wicked problems requires purposeful problem-solving approaches that involve cooperation and ownership from a broad range of stakeholders across multiple disciplines (7). Deliberative democracy is one form of problem-solving in which diverse groups of citizens gather, think critically, and generate solutions together (7). An essential component of deliberative democracy is the “deliberative inquiry” or “social learning” phase that, ideally, should come first and be separated from decision making (7, 8). While the deliberative inquiry phase is not in and of itself sufficient to resolve wicked problems, it can be a critical starting point. The purpose of this phase is to build a foundation of trust and understanding in which participants can learn from one another and begin to see each other as “collaborators facing a wicked problem” without pressure to reach an agreement (7, 8). Whereas polarization dismantles democracy by encouraging people to be more obstructionist and less deliberative (9), dialogue has been found to build understanding, integrate knowledge, and reduce fear and anger among diverse groups of stakeholders on difficult, interdisciplinary public problems (10). When people with opposing views can engage openly in dialogue, they have increased opportunities to work towards consensual problem solving by jointly defining problems and creatively identifying and implementing solutions that are more likely to be acceptable to various stakeholders with competing interests (11).

As institutions designed for learning and growth, universities are an ideal setting to serve as “critical spaces” in which students and scholars participate in deliberative inquiry on shared problems (12, 13). This can occur through a structured process of open and respectful dialogue where participants consider knowledge from various disciplines along with the underlying values that inform their views (12, 13).

Several organizations, primarily outside the field of public health, have developed structured approaches to creating space for deliberative inquiry through dialogue. Reflective Structured Dialogue (RSD) is a well-known method whose purpose is not to find solutions but to build a foundation of mutual understanding, empathy, and trust between participants upon which future solutions can be developed (14–18). Storytelling is a key ingredient in RSD, allowing participants to build trust and to offer differing points of view without being contentious (17, 19). RSD relies on careful preparation to craft conversation agreements (i.e., ground rules) and thought-provoking questions that facilitators use to encourage participants to reflect on and share their personal experiences related to the discussion topics. A shared commitment to the conversation agreements helps create an environment supportive of active listening, open-mindedness, and authenticity where participants can learn from one another. Due to this carefully designed structure, RSD is uniquely suited to handle uneasiness and moments of disruption (18, 20).

While extensive research has documented the positive effects of various types of dialogue on polarization and conflict reduction, rigorous studies specific to RSD are more limited; however, the existing evidence related to RSD is promising. For example, a qualitative study showed that RSD can create and deepen positive connections, increase self-reflection and growth, improve communication skills, and help people understand different perspectives and viewpoints (15). Another case study found that RSD helped build trust and strengthen relationships between people that had opposing viewpoints on LGBTQ+ inclusion in a university setting (21).

Recognizing the scarcity of scientific evidence and the potential value of RSD in resolving wicked problems, the primary aims of this study were to assess the effects of participation in RSD—specifically about divisive public health-related topics—on university students’ polarization-related attitudes. The study also sought to understand students’ opinions about their participation in the RSD experience.

## 2. Materials and methods

### 2.1. Project description

In collaboration with Living Room Conversations, the Brigham Young University (BYU) Campus Conversations project was developed to create opportunities for dialogue around divisive public health-related issues among BYU students, with the objective of increasing mutual understanding, respect, and empathy in the campus community. The project was funded through a grant from the Heterodox Academy, a nonpartisan nonprofit organization whose mission is “to improve the quality of research and education in universities by increasing open inquiry, viewpoint diversity, and constructive disagreement” (22).

The project used an RSD approach to hold structured conversations about three specific public health topics chosen based on their relevance to the BYU community: COVID-19, mental health, and racism. The project team partnered with Living Room Conversations to create separate structured conversation guides for each of the three topics (see Supplementary material). The guides were tailored to the specific needs of the target population; for example, the conversation guide addressing racism was titled, “Race and Faith,” and was designed to encourage students to reflect on their race-related beliefs and experiences at BYU, a religious institution where most students are members of The Church of Jesus Christ of Latter-day Saints. Each conversation guide was designed to last 90 min and included the following elements:

1. Introductions: participants share their names and why they chose to participate.
2. Conversation agreements: facilitator and participants read through a list of conversation agreements which all participants are asked to agree to follow before proceeding.
3. Getting to know each other: participants select a question from a list of introductory questions and take 2 minutes each to share their responses to help them introduce themselves to the rest of the group.
4. Exploring the topic: the facilitator reads a short paragraph introducing participants to the conversation topic. Participants (including the facilitator) take turns selecting a question from a list of topic-related questions and take 2 minutes each to share their responses with the group. After everyone has a chance to share, participants ask and answer follow-up questions. If time permits, participants repeat this process by selecting another question from the list.
5. Reflecting on the conversation: participants select a question from a list of final reflection questions and take 2 minutes each to share their responses with the group.
6. Closing: the facilitator thanks participants for their participation and reminds them to complete the post-conversation survey.

Table 1 summarizes key features of the different types of conversations. All conversations took place in small groups of four to seven people, including one to two student facilitators. Each student facilitator was a member of the project team and had received training from Living Room Conversations to guide the conversations. Some of the conversations were held in a large conference room (referred to here as “large group conversations”) with three or more conversations taking place at the same time in separate small groups. Other conversations took place in smaller conference rooms (referred to here as “medium group conversations”) with two concurrent separate conversations taking place at the same time in separate small groups. The rest of the conversations were held in small classrooms or homes (referred to here as “small group conversations”) with only one conversation happening at a time. Different conversation types and sizes were used to assess whether participant outcomes differed by conversation features.

**Table 1:** Conversation features.

	Size of conversation group		
	Small group	Medium group	Large group
Conversation location	Small classrooms or homes	Small conference rooms	Large conference rooms
# of simultaneous conversations of 4–7 People	1	2	3 or more
Topic	Racism	COVID-19 or mental health	COVID-19 or mental health
One-time or series of 3	Series of 3	One-Time	One-Time

The large- and medium-group conversations addressed either COVID-19 or mental health while the small group conversations addressed the topic of racism. The large- and medium-group conversations required participants to attend only one conversation, but the small group racism conversations were conducted over a series of three conversations with the same group of participants, requiring the participants to attend all three conversations. Separate conversation guides on racism were developed and used for each of the conversations in the racism series. While most of the conversations were held in person on or near BYU’s campus, two were held virtually via Zoom due to scheduling conflicts. Different conversation topics were used to assess whether participant outcomes differed by topic.

### 2.2. Participants

The project team consisted of two public health faculty, two public health graduate student project managers, and 12 public health undergraduate student facilitators. Conversation participants were current and recently graduated (within 1 year) BYU undergraduate and graduate students. Conversation participants were recruited in multiple ways, including via information booths, advertisements on university clubs’ social media accounts, informational flyers, classroom announcements, and word-of-mouth.

### 2.3. Data collection and analysis

The project team developed the pre- and post-conversation surveys based on reviews of gray and academic literature on RSD-related activities. In addition to basic demographic questions, the surveys primarily used (with some modifications) previously tested questions from the Social Cohesion Impact Measure (SCIM) to measure outcomes related to participants’ polarization attitudes towards other groups as well as to participants “bridging” mindsets and attitudes. The seven outcomes assessed in the survey included: openness (i.e., to new information and understanding), tribal identity (i.e., identity with one’s group relative to other groups), intellectual humility (i.e., willingness to be wrong), respect (i.e., appreciation for differing opinions), empathy (i.e., ability to see things from other people’s perspectives), animosity (i.e., negative feelings towards others), and moral disdain (i.e., perception that others are morally inferior). In total, 23 attitude questions with five-point Likert scale response options were included in both the pre- and post-conversation surveys. For most questions, the “preferred” answer was Strongly Agree; however, five questions were worded differently so that Strongly Disagree was the “preferred” response. The post-conversation survey also included 10 total Likert scale and open-ended questions regarding participants’ overall experience with the conversations.

All participants were invited to complete the pre- and post-conversations surveys electronically using Qualtrics online survey software. After participants signed up to join a conversation, they received an email with a link to the pre-conversation survey. Participants were also given time to complete the survey at the beginning of the conversation if they had not previously done so. The post-conversation survey link was emailed to participants within a week following the conversation. To preserve anonymity, pre- and post-surveys were linked through unique participant IDs, and no personal information was tied to survey results. All participants who completed the pre- and post-surveys received a $10 Amazon gift card.

The research team used Microsoft Excel to organize and analyze the data. Summary statistics for the demographic questions and each of the Likert questions were calculated, and two-tailed paired t-tests were conducted to identify statistically significant differences between the pre- and post-conversations surveys.

## 3. Results

A total of 139 study participants partook in one or more of 27 conversations. Of the participants, 118 completed both surveys and were included in the analysis; 108 were student participants, and the other 10 were student facilitators. Table 2 summarizes key demographic variables of the included participants. Ages ranged from 18 to 29 years old, with a mean of 22.4 years old. Most participants were either seniors (42.3%) or juniors (28%). There was a nearly equal split between male and female participants, and most participants (87.3%) identified as straight/heterosexual. Most participants were single (65.3%), followed by married (28.8%), engaged (5.1%), and divorced (0.9%).

**Table 2:** Participant demographics (*n* = 118).

	Count	Percentage
Gender		
Male	58	49.2
Female	60	50.9
Age		
18–20	17	14.5
21–23	74	62.7
24–26	20	17
27–29	7	6
Sexual orientation		
Heterosexual	103	87.3
LGBTQ+	10	8.7
Questioning	5	4.2
Religious affiliation		
The Church of Jesus Christ of Latter-day Saints	116	98.3
Agnostic	2	1.7
Political views		
Very liberal	2	1.7
Liberal	13	11
Moderately liberal	27	22.9
Moderately conservative	42	35.6
Conservative	21	17.8
Libertarian	6	5.1
Other	7	5.9
Race/ ethnicity		
White	100	69.9
Asian American/Asian	17	11.9
Hispanic or Latino/a	14	9.8
African American/Black	4	2.1
Native American/American Indian	2	1.4
Other	6	4.2
Current status in school		
Freshman	5	4.2
Sophomore	23	19.5
Junior	33	28
Senior	50	42.4
Graduate Student	7	6
Relationship status		
Not married or engaged	78	66.2
Engaged	6	5.1
Married	34	28.8

Most participants (98.3%) indicated their religion as The Church of Jesus Christ of Latter-day Saints, the sponsoring religious institution of BYU. Most participants identified as White (69.9%). The second largest race/ethnicity represented was Asian American/Asian (11.9%), followed by Hispanic/Latino (6.3%), and Other (4.2%). Project participants were divided across the political spectrum. The greatest percentage of participants defined themselves as Moderately Conservative (35.6%), followed by Moderately Liberal (22.9%), Conservative (17.8%), and Liberal (11.0%).

Overall, the conversations appeared to be effective in improving participants’ polarization and bridging attitudes. Tables 3, 4 show that 19 of 23 pre- and post-survey questions showed increases in the “preferred” response (Strongly Agree or Strongly Disagree, depending on question wording), and the pre-post differences for four of the 19 questions were statistically significant (*p* < 0.05).

**Table 3:** Strongly agree preferred statements.

Statement (outcome)	Difference between pre- and post-surveys (%)				*p* value *p < 0.05
	Disagree (somewhat and strongly)	Neither agree nor disagree	Somewhat agree	Strongly agree	
I enjoy having conversations with people holding differing opinions or perspectives (Openness)	−1.7	−5.9	−8.4	+16.0	0.026*
I am comfortable having conversations with people of different opinions (Openness)	−2.5	2.5	−14.3	+14.3	0.073*
If people different than me are praised it makes me feel good (Tribal Identity)	−1.7	−14.3	2.5	+13.5	0.010*
I can admit when my opinions or perspectives could be wrong (Intellectual Humility)	0.8	1.7	−13.5	+10.9	0.385
I respect differing opinions even when I do not agree (Respect)	0	−1.7	−6.7	+8.4	0.250
I can often see things from differing points of view (Empathy)	−0.8	0	−6.7	+7.6	0.240
I am comfortable having conversations with people of different political orientations (Animosity)	−3.4	−1.7	−1.7	+6.7	0.107
If I met someone who is different than me, I’d feel connected to this person (Tribal Identity)	1.7	−6.7	−0.8	+5.9	0.443
It’s important to understand different people by imagining how things look from their perspective (Empathy)	0.8	−0.8	−5	+5.0	0.524
I am comfortable speaking with people different than me (Animosity)	−2.5	3.4	−5	+4.2	0.391
I am comfortable having conversations with people of different sexual orientations (Animosity)	−1.7	4.2	−5	+2.5	0.786
I am comfortable being around people different than me (Animosity)	0	−0.8	−1.7	+2.5	0.702
I am comfortable having conversations with people of different gender orientations (Animosity)	−1.7	5.9	−4.2	+0.0	0.862
I am comfortable having friends who are different than me (Animosity)	0	1.7	−0.8	−0.8	0.840
I would say that people different than me are generally good people (Moral Disdain)	−0.8	−0.8	3.4	−1.7	0.815
I am comfortable having conversations with people of a different race (Animosity)	−0.8	0	4.2	−3.4	0.885

**Table 4:** Strongly disagree preferred statements.

Survey question (outcome)	Difference between pre- and post-surveys (%)				*p* value *p < 0.05
	Agree (somewhat and Strongly)	Neither agree nor disagree	Somewhat disagree	Strongly disagree	
I consider people with differing opinions to be deceived (Moral Disdain)	−6.7	−10.1	5	+11.8	0.018*
I consider people with differing opinions to be uneducated (Moral Disdain)	−3.4	−3.4	−5	+11.8	0.103
I consider people with differing opinions to be misinformed (Moral Disdain)	0	−12.6	2.5	+10.1	0.165
I become angry when thinking about different people (Empathy)	3.4	−0.8	−9.2	+6.7	0.833
In a typical conversation I do the majority of the talking (Respect)	9.2	−9.2	−4.2	+4.2	0.591
I find it difficult to see things from a different point of view (Empathy)	−2.5	0	−0.8	+3.4	0.760
I consider people with differing opinions to be wrong (Intellectual Humility)	−0.8	0.8	−3.4	+3.4	1.00

The greatest statistically significant improvements between the pre- and post-conversation surveys were seen in the two questions related to the outcome of openness. Before the conversation, 42.9% of participants strongly agreed with the statement, “I enjoy having conversations with people holding differing opinions or perspectives”; after the conversation 58.8% of participants strongly agreed. Similarly, before the conversation, 52.1% of participants strongly agreed with the statement, “I am comfortable having conversations with people of different opinions”; after the conversation 66.4% strongly agreed.

The two other outcomes showing statistically significant improvements between the pre- and post-surveys were tribal identity and moral disdain. For tribal identity, before the conversation 18.6% of participants strongly agreed with the statement, “If people different than me are praised it makes me feel good,” compared to 32.2% after the conversation. For moral disdain, before the conversation 19.5% of participants strongly disagreed with the statement, “I consider people with different opinions to be deceived,” compared to 31.4% after the conversation. Questions measuring each of the other outcomes showed improvements between the pre- and post-surveys, but these improvements were not statistically significant.

Among the four questions showing either no change or a decrease in the preferred response between the pre- and post-conversation surveys, three measured the outcome of animosity while one measured the outcome of moral disdain. None of the pre-post differences for these questions were statistically significant. The greatest negative change (−3.4%) was for the statement, “I am comfortable having conversations with people of a different race.” Before the conversation, 84% of participants strongly agreed compared to 80.7% after the conversation.

The results from the questions unique to the post-conversation survey (see Table 5) further indicated the successful nature of the conversations. Over 50% of participants strongly agreed with seven of the ten unique post-survey statements. The most positive result was regarding the conversation being a positive experience, with 81.5% of participants strongly agreeing and 15.1% somewhat agreeing. The second most positive result was regarding the conversation helping participants understand the experience of others, with 78.2% of participants strongly agreeing and 18.5% somewhat agreeing.

**Table 5:** Post survey only statements.

Statement	% disagree	% neither agree nor disagree	% somewhat agree	% strongly agree
Participation in BYU campus conversations has been a positive experience	0.8	1.7	15.1	81.5
Participation in BYU campus conversations has helped me understand the experiences of others	0	2.5	18.5	78.2
Participation in BYU campus conversations has helped me feel more connected to others	0.8	6.7	27.7	63.9
Participation in BYU campus conversations has helped me gain new perspectives	0.8	6.7	28.6	63.0
Participation in BYU campus conversations has increased my compassion or empathy for others	1.7	6.7	27.7	63.0
Participation in BYU campus conversations has helped me see important issues from a different point of view	4.2	5.9	31.1	58.0
Participation in BYU campus conversations has increased my appreciation for perspectives different than my own	0.0	9.2	37.8	52.1
Participation in BYU campus conversations has increased my confidence in sharing personal experiences and perspectives	3.4	9.2	37	49.6
Participation in BYU campus conversations has increased my hope for the future	1.7	18.5	31.1	47.9
Participation in BYU campus conversations has increased my trust in others	3.4	16.8	35.3	43.7

The overall results were similar when comparing participant responses between different conversation types (series, non-series), topics (COVID-19, mental health, racism), and meeting sizes (small, medium, large). Few statistically significant differences were identified between these different conversation variations, which may be attributed to the smaller sample sizes of participants for each variation.

## 4. Discussion

This study shows that RSD is positively associated with improved polarization-related attitudes among university students. While not all changes were statistically significant, most survey questions showed positive changes between the pre- and post-surveys, with the biggest differences coming in questions related to outcomes of openness, tribal identity, and moral disdain. Nearly all students believed their experiences with RSD were positive, helped them understand the experiences of and feel more connected to others, and increased their empathy and compassion. Bethel and colleagues, who likewise employed the Living Room Conversations and RSD models in a study involving students from two different institutions, similarly reported that student participants found conversations to be meaningful and effective at increasing empathy, connection, and in providing insight into the lived experiences of others (23).

When asked to comment on their overall experiences, the open-ended responses from participants supported the quantitative findings. A representative comment from one student said, “I enjoyed being in a setting where people are encouraged to listen to others first without rebutting them. I usually do not talk about these kinds of topics due to being anxious about negative responses.” Several students mentioned their desire to incorporate structured conversations into the university campus community in more permanent ways. For example, one participant wrote, “I believe this is important enough to be included in curriculum. I think that every BYU student should participate in such a discussion before they graduate.”

Together, the quantitative data and participant comments suggest that, as in previous studies, RSD helped participants create connections and understand different perspectives (15, 21). This study also helps demonstrate how deliberative inquiry can contribute to student learning in higher education setting (12). These empirical findings are useful in supporting the theoretical justifications made about the value of RSD and other types of dialogue in building trust and understanding between diverse groups as a prerequisite to problem-solving (16–18). Successful consensual problem solving for wicked problems requires dialogue to create the conditions in which stakeholders with distinct interests can collaborate towards progress on solutions (11).

A potential limitation to this project is the lack of diversity among participants. Most participants identified as members of The Church of Jesus Christ of Latter-day Saints, as White/Caucasian, and as heterosexual. This demographic makeup was anticipated due to the demographics of BYU; however, such demographics should be considered when interpreting the project results and when seeking to generalize them to larger populations. A second potential limitation regarding generalizability is that this project was done with only university students that were to a large degree self-selected into the study. While the number and type of participants was chosen based on logistical concerns, a larger, more diverse sample in future projects would help increase confidence in the results and could potentially yield additional insights about conversation sizes, topics, etc. that were not observed in this project.

Structured dialogues, such as those used in this study, are more important now than ever as society becomes increasingly polarized along ideological, religious, racial, and other divides. While this is a relatively small demonstration project in a somewhat unique context, the results are promising and lend strength to the argument that RSD can serve as a useful tool in university settings and in the larger context of addressing wicked public health problems in communities. Future projects should explore additional applications of RSD and use rigorous evaluation methods to better understand how to optimize outcomes.

## Data availability statement

The raw data supporting the conclusions of this article will be made available by the authors, without undue reservation.

## Ethics statement

The studies involving humans were approved by Brigham Young University Institutional Review Board. The studies were conducted in accordance with the local legislation and institutional requirements. The ethics committee/institutional review board waived the requirement of written informed consent for participation from the participants or the participants’ legal guardians/next of kin because Study posed minimal to no risk to participants.

## Author contributions

CW, JB, PH, and JG contributed to conception and design of the study. CW, JB, LB, PC, SD, and MF recruited participants and collected data. CW performed the statistical analysis. CW and JB wrote the first draft of the manuscript. All authors contributed to the article and approved the submitted version.
