# Supplementary material for: Reflective structured dialogue as a tool for addressing wicked public health problems
Source: Front Public Health. 2023 Sep 25;11:1220029. doi: 10.3389/fpubh.2023.1220029 (PMC10560707; doi:10.3389/fpubh.2023.1220029)
Supplement: Supplementary file 1 [file Data_Sheet_1.pdf]

Conversation Guide: Mental Health

Living Room Conversations offers a simple, sociable and structured way to practice communicating across differences while building understanding and relationships. Typically, 4-6 people meet in person or by video call for about 90 minutes to listen to and be heard by others on one of our nearly 100 topics. Rather than debating or convincing others, we take turns talking to share, learn, and be curious. No preparation is required, though background links with balanced views are available on some topic pages online. Anyone can host using these italicized instructions. Hosts also participate.

I. Introductions: Why We're Here (~10 minutes)

Each participant has 1 minute to introduce themselves.

- Share your name, where you live, what drew you here, and if this is your first conversation.

II. Conversation Agreements: How We'll Engage (~5 minutes)

These will set the tone of our conversation; participants may volunteer to take turns reading them aloud.

- **Be curious and listen to understand.** Conversation is as much about listening as it is about talking. You might enjoy exploring how others' experiences have shaped their values and perspectives.
- **Show respect and suspend judgment.** People tend to judge one another. Setting judgment aside opens you up to learning from others and makes them feel respected and appreciated. Try to truly listen, without interruption or crosstalk.
- **Note any common ground as well as any differences.** Look for areas of agreement or shared values that may arise and take an interest in the differing beliefs and opinions of others.
- **Be authentic and welcome that from others.** Share what's important to you. Speak from your experience. Be considerate of others who are doing the same.
- **Be purposeful and to the point.** Do your best to keep your comments concise and relevant to the question you are answering. Be conscious of sharing airtime with other participants.
- **Own and guide the conversation.** Take responsibility for the quality of your participation and the conversation as a whole. Be proactive in getting yourself and others back on track if needed. Use an agreed upon signal like the "time out" sign if you feel the agreements are not being honored.

III. Question Rounds: What We'll Talk About

Optional: a participant can keep track of time and gently let people know when their time has elapsed.

Round One: Getting to Know Each Other (~10 min)

Each participant can take 1-2 minutes to answer one of these questions:

- What are your hopes and concerns for your family, community and/or the country?
- What would your best friend say about who you are?
- What sense of purpose / mission / duty guides you in your life?

This is an open source project. Please use, share and modify with attribution to LivingRoomConversations.org

Round Two: Exploring the Topic -- Mental Health (~40 min)

One participant can volunteer to read this paragraph.

As an institution, BYU prepares its students to serve others, see the connection between the gospel and what is taught in classrooms, and be distinct in upholding standards. These benchmarks can enrich student life and connect students to a higher purpose. There are also pressures and questions that stem from campus culture and can directly impact the mental and emotional health of students like a tendency toward perfectionism and accompanying sense of guilt if an individual doesn't measure up, the pressure of dating, getting married, and starting a family and what that means for LGBTQ+ students. With all of this in play, how do we care for ourselves and each other?

In this conversation, we invite you to step back from the pressures you may have in your life, connect with others around how mental health impacts each of you, and find strength in the thoughts and experiences you share.

Take ~2 minutes each to answer a question below without interruption or crosstalk. After everyone has answered, the group may take a few minutes for clarifying or follow up questions/responses. Continue exploring additional questions as time allows.

- How do religion, culture, family, or parts of your identity influence how you think about mental health and mental health interventions?
- What is the impact of mental health issues on campus and in your community? Have you seen changes in these issues over time?
- This is the third year COVID is actively affecting schools. What has been difficult for you? What have you learned and appreciated? What has surprised you?
- Do you find yourself caring for or being affected by the mental and emotional health of others? What does that look like and what impact does it have on you?
- What influence or impact does social media have on you? Do you use or avoid it? What do you like and/or dislike about social media?
- How do you care for or consider your mental and emotional health? What is the impact of that?

Round Three: Reflecting on the Conversation (~15 min)

Take 2 minutes to answer one of the following questions:

- What was most meaningful / valuable to you in this Living Room Conversation?
- What learning, new understanding or common ground was found on the topic?
- How has this conversation changed your perception of anyone in this group, including yourself?
- Is there a next step you would like to take based upon the conversation you just had?

Closing (~5 min)

- *Give us feedback!* Use livingroomconversations.org/feedback-form/ or QR code
- *Donate!* Make more of these possible; give at livingroomconversations.org/donate/
- *Join or host more conversations!* With a) this group by exchanging your emails; b) others in person and/or by video call online. Get more involved or learn how to host at livingroomconversations.org/get-involved/

This is an open source project. Please use, share and modify with attribution to LivingRoomConversations.org
