# Supplementary material for: Reflective structured dialogue as a tool for addressing wicked public health problems
Source: Front Public Health. 2023 Sep 25;11:1220029. doi: 10.3389/fpubh.2023.1220029 (PMC10560707; doi:10.3389/fpubh.2023.1220029)
Supplement: Supplementary file 2 [file Data_Sheet_2.pdf]

Conversation Guide: Public Health

Living Room Conversations offers a simple, sociable and structured way to practice communicating across differences while building understanding and relationships. Typically, 4-7 people meet in person or by video call for about 90 minutes to listen to and be heard by others on one of our nearly 100 topics. Rather than debating or convincing others, we take turns talking to share and learn, and be curious. No preparation is required, though background links with balanced views are available on some topic pages online. *Anyone can host using these italicized instructions. Hosts also participate.*

Introductions: Why We're Here (~10 minutes)

Each participant has 1 minute to introduce themselves.

- Share your name, where you live, what drew you here, and if this is your first conversation.

Conversation Agreements: How We'll Engage (~5 minutes)

These will set the tone of our conversation; participants may volunteer to take turns reading them aloud.

- **Be curious and listen to understand.** Conversation is as much about listening as it is about talking. You might enjoy exploring how others' experiences have shaped their values and perspectives.

- **Show respect and suspend judgment.** People tend to judge one another. Setting judgement aside opens you up to learning from others and makes them feel respected and appreciated. Try to truly listen, without interruption or crosstalk.

- **Note any common ground as well as any differences.** Look for areas of agreement or shared values that may arise and take an interest in the differing beliefs and opinions of others.

- **Be authentic and welcome that from others.** Share what's important to you. Speak from your experience. Be considerate of others who are doing the same.

- **Be purposeful and to the point.** Do your best to keep your comments concise and relevant to the question you are answering. Be conscious of sharing airtime with other participants.

- **Own and guide the conversation.** Take responsibility for the quality of your participation and the conversation as a whole. Be proactive in getting yourself and others back on track if needed. Use an agreed upon signal like the "time out" sign if you feel the agreements are not being honored.

Question Rounds: What We'll Talk About

Optional: a participant can keep track of time and gently let people know when their time has elapsed.

Round One: Getting to Know Each Other (~10 min)

Each participant can take 1-2 minutes to answer one of these questions:

- What are your hopes and concerns for your family, community and/or the country?
- What would your best friend say about who you are?
- What sense of purpose / mission / duty guides you in your life?

This is an open source project. Please use, share and modify with attribution to LivingRoomConversations.org

Round Two: Exploring the Topic -- Public Health (~40 min)

One participant can volunteer to read this paragraph.

Most people would agree that a healthy population is a shared societal goal. With the global reach and impact of COVID-19, the policies, intentions, and concerns of public health officials (and the entire field) have been in the news and under scrutiny. Governments, communities, and college campuses around the world have scrambled to come up with policies that would support overall public health only to get backlash for doing too much or too little, being too late or too early. Mask mandates, closing businesses, and social distancing have evoked a spectrum of responses and frustrations from citizens. The role of public health is to take action to improve the health of populations and groups. What does it look like to prioritize the collective needs of the communities we live in while honoring individuals' beliefs and the freedom to make our own health choices?

Take ~2 minutes each to answer a question below without interruption or crosstalk. After everyone has answered, the group may take a few minutes for clarifying or follow up questions/responses. Continue exploring additional questions as time allows.

- Where do you get your approach to health from? (friends, family, doctors, social media, etc.)
- What has been your experience with public health mandates during the pandemic? What did you appreciate? struggle with?
- What informs your decision around vaccines? What do you wish others understood about your decision?
- Public health involves trust on different levels-- between members of the community, institutions, governments. When it comes to public health issues, who do you trust and why? Where do you find information to be better informed?
- Have you experienced any judgment or assumptions based on whether or not you wear a mask in public? Have you judged or made assumptions about others based on their decision whether or not to wear a mask?
- What is the right balance between the collective needs of the communities we live in and the freedom to make our own individual health choices?

Round Three: Reflecting on the Conversation (~15 min)

Take 2 minutes to answer one of the following questions:

- What was most meaningful / valuable to you in this Living Room Conversation?
- What learning, new understanding or common ground was found on the topic?
- How has this conversation changed your perception of anyone in this group, including yourself?
- Is there a next step you would like to take based upon the conversation you just had?

Closing (~5 min)

- *Give us feedback!* Use livingroomconversations.org/feedback-form/ or QR code
- *Donate!* Make more of these possible; give at livingroomconversations.org/donate/
- *Join or host more conversations!* With a) this group by exchanging your emails; b) others in person and/or by video call online. Get more involved or learn how to host at livingroomconversations.org/get-involved/

Thank you!

This is an open source project. Please use, share and modify with attribution to LivingRoomConversations.org
