# Supplementary material for: Reflective structured dialogue as a tool for addressing wicked public health problems
Source: Front Public Health. 2023 Sep 25;11:1220029. doi: 10.3389/fpubh.2023.1220029 (PMC10560707; doi:10.3389/fpubh.2023.1220029)
Supplement: Supplementary file 3 [file Data_Sheet_3.pdf]

Race and Faith Communities Series: Listening Courageously

Living Room Conversations offers a simple, sociable and structured way to practice communicating across differences while building understanding and relationships. Typically, 4-7 people meet in person or by video call for about 90 minutes to listen to and be heard by others on one of our nearly 100 topics. Rather than debating or convincing others, we take turns talking to share and learn, and be curious. No preparation is required, though background links with balanced views are available on some topic pages online. *Anyone can host using these italicized instructions. Hosts also participate.*

Introductions: Why We're Here (~10 minutes)

Each participant has 1 minute to introduce themselves.

- Share your name, where you live, what drew you here, and if this is your first conversation.

Conversation Agreements: How We'll Engage (~5 minutes)

These will set the tone of our conversation; participants may volunteer to take turns reading them aloud.

- **Be curious and listen to understand.** Conversation is as much about listening as it is about talking. You might enjoy exploring how others' experiences have shaped their values and perspectives.
- **Show respect and suspend judgment.** People tend to judge one another. Setting judgement aside opens you up to learning from others and makes them feel respected and appreciated. Try to truly listen, without interruption or crosstalk.
- **Note any common ground as well as any differences.** Look for areas of agreement or shared values that may arise and take an interest in the differing beliefs and opinions of others.
- **Be authentic and welcome that from others.** Share what's important to you. Speak from your experience. Be considerate of others who are doing the same.
- **Be purposeful and to the point.** Do your best to keep your comments concise and relevant to the question you are answering. Be conscious of sharing airtime with other participants.
- **Own and guide the conversation.** Take responsibility for the quality of your participation and the conversation as a whole. Be proactive in getting yourself and others back on track if needed. Use an agreed upon signal like the "time out" sign if you feel the agreements are not being honored.

Question Rounds: What We'll Talk About

Optional: a participant can keep track of time and gently let people know when their time has elapsed.

Round One: Getting to Know Each Other (~10 min)

Each participant can take 1-2 minutes to answer one of these questions:

- What are your hopes and concerns for your family, community and/or the country?
- What would your best friend say about who you are?
- What sense of purpose / mission / duty guides you in your life?

Round Two: Exploring the Topic - Race & Faith Communities: Listening Courageously (~40 min)

One participant can volunteer to read this paragraph.

Dr. Maya Angelou said, "Courage is the most important of all virtues, because without courage you cannot practice any of the other virtues consistently." When you think of being courageous, you might think of facing a common fear like the fear of heights or public speaking. Facing fears like these involves discomfort and uncertainty. We might wonder if the potential benefits will outweigh the potential risks. But why might it take courage to listen as we embark on having conversations around race? The Latin root for courage comes from the word for heart. How will you stay connected to your heart if the conversation becomes uncomfortable? This conversation will establish the groundwork for being able to share personal experiences around the intersection of race and faith in our second conversation.

Take ~2 minutes each to answer a question below without interruption or crosstalk. After everyone has answered, the group may take a few minutes for clarifying or follow up questions/responses. Continue exploring additional questions as time allows.

- In the context of talking about race, what does "courageous listening" mean for you? Consider times you have listened, or been listened to in this way. What happened?
- What fears, if any, do you have about how you will feel or what you will say or do when listening to others during this conversation series?
- How will you hang in there when something is hard to hear? How will you support yourself and others if something is said that creates tension or is potentially hurtful?
- What are your hopes for these conversations? What are the potential benefits you might receive from listening to others with differing beliefs or experiences?

Round Three: Reflecting on the Conversation (~15 min)

Take 2 minutes to answer one of the following questions:

- What was most meaningful / valuable to you in this Living Room Conversation?
- What learning, new understanding or common ground was found on the topic?
- How has this conversation changed your perception of anyone in this group, including yourself?
- Is there a next step you would like to take based upon the conversation you just had?

Closing (~5 min)

- *Give us feedback!* Use livingroomconversations.org/feedback-form/ or QR code
- *Donate!* Make more of these possible; give at livingroomconversations.org/donate/
- *Join or host more conversations!* With a) this group by exchanging your emails; b) others in person and/or by video call online. Get more involved or learn how to host at livingroomconversations.org/get-involved/

Thank you!

This is an open source project. Please use, share and modify with attribution to LivingRoomConversations.org
