# Supplementary material for: Reflective structured dialogue as a tool for addressing wicked public health problems
Source: Front Public Health. 2023 Sep 25;11:1220029. doi: 10.3389/fpubh.2023.1220029 (PMC10560707; doi:10.3389/fpubh.2023.1220029)
Supplement: Supplementary file 4 [file Data_Sheet_4.pdf]

LivingRoom Conversations

Respect. Relate. Connect.

Race and Faith Communities Series: The Way Forward

Living Room Conversations offers a simple, sociable and structured way to practice communicating across differences while building understanding and relationships. Typically, 4-7 people meet in person or by video call for about 90 minutes to listen to and be heard by others on one of our nearly 100 topics. Rather than debating or convincing others, we take turns talking to share and learn, and be curious. No preparation is required, though background links with balanced views are available on some topic pages online. *Anyone can host using these italicized instructions. Hosts also participate.*

Introductions: Why We're Here (~10 minutes)

Each participant has 1 minute to introduce themselves.

- Share your name, where you live, what drew you here, and if this is your first conversation.

Conversation Agreements: How We'll Engage (~5 minutes)

These will set the tone of our conversation; participants may volunteer to take turns reading them aloud.

- **Be curious and listen to understand.** Conversation is as much about listening as it is about talking. You might enjoy exploring how others' experiences have shaped their values and perspectives.
- **Show respect and suspend judgment.** People tend to judge one another. Setting judgement aside opens you up to learning from others and makes them feel respected and appreciated. Try to truly listen, without interruption or crosstalk.
- **Note any common ground as well as any differences.** Look for areas of agreement or shared values that may arise and take an interest in the differing beliefs and opinions of others.
- **Be authentic and welcome that from others.** Share what's important to you. Speak from your experience. Be considerate of others who are doing the same.
- **Be purposeful and to the point.** Do your best to keep your comments concise and relevant to the question you are answering. Be conscious of sharing airtime with other participants.
- **Own and guide the conversation.** Take responsibility for the quality of your participation and the conversation as a whole. Be proactive in getting yourself and others back on track if needed. Use an agreed upon signal like the "time out" sign if you feel the agreements are not being honored.

Question Rounds: What We'll Talk About

Optional: a participant can keep track of time and gently let people know when their time has elapsed.

Round One: Getting to Know Each Other (~10 min)

Each participant can take 1-2 minutes to answer one of these questions:

- Have you told others about participating in these conversations? Why or why not?
- Is there anything you are dwelling on or grappling with after the last conversation?
- Has participating in these conversations affected the way you listen to others discuss race?

This is an open source project. Please use, share and modify with attribution to LivingRoomConversations.org

Round Two: Exploring the Topic -- Race and Faith Communities: The Way Forward (~40 min)

One participant can volunteer to read this paragraph.

The last two conversations have focused on bringing our hearts to conversations around race and building understanding around race in our faith communities. This conversation invites us to look forward, to consider the promises of our faith community while reflecting on what we've learned through this process.

Take ~2 minutes each to answer a question below without interruption or crosstalk. After everyone has answered, the group may take a few minutes for clarifying or follow up questions/responses. Continue exploring additional questions as time allows.

- What is the promise of our faith community to its members? To the surrounding community?
- How are we living up to our promise to each other and to our faith promises when it comes to ensuring we and our societies are treating all human beings with the respect and care we would want for ourselves?
- Is there anything you've been wanting to ask another member of the group but did not feel comfortable doing so? Are you willing to ask that question now?
- How has participation in this cohort shifted your perception of your own personal racial, ethnic, or spiritual narrative?
- What would a faith community that values racial and ethnic differences look like? What hopes and fears come up for you thinking about that faith community?
- If you were creating a reconciling agenda for change to unite and energize our community, what would it include?

Round Three: Reflecting on the Conversation (~15 min)

Take 2 minutes to answer one of the following questions:

- What was most meaningful / valuable to you in this conversation series?
- How has your understanding evolved or shifted over the course of these conversations?
- Are there any resources that you would like to share with the group to support their efforts in creating capacity for these types of conversations?
- Are there any next steps you plan to take individually or would like to do as a group?

Closing (~5 min)

- *Give us feedback!* Use livingroomconversations.org/feedback-form/ or QR code
- *Donate!* Make more of these possible; give at livingroomconversations.org/donate/
- *Join or host more conversations!* With a) this group by exchanging your emails; b) others in person and/or by video call online. Get more involved or learn how to host at livingroomconversations.org/get-involved/

Thank you!

This is an open source project. Please use, share and modify with attribution to LivingRoomConversations.org
