# Supplementary material for: Reflective structured dialogue as a tool for addressing wicked public health problems
Source: Front Public Health. 2023 Sep 25;11:1220029. doi: 10.3389/fpubh.2023.1220029 (PMC10560707; doi:10.3389/fpubh.2023.1220029)
Supplement: Supplementary file 5 [file Data_Sheet_5.pdf]

Race and Faith Communities Series: Conversation 3

Living Room Conversations offers a simple, sociable and structured way to practice communicating across differences while building understanding and relationships. Typically, 4-7 people meet in person or by video call for about 90 minutes to listen to and be heard by others on one of our nearly 100 topics. Rather than debating or convincing others, we take turns talking to share and learn, and be curious. No preparation is required, though background links with balanced views are available on some topic pages online. *Anyone can host using these italicized instructions. Hosts also participate.*

Introductions: Why We're Here (~10 minutes)

Each participant has 1 minute to introduce themselves.

- Share your name, where you live, what drew you here, and if this is your first conversation.

Conversation Agreements: How We'll Engage (~5 minutes)

These will set the tone of our conversation; participants may volunteer to take turns reading them aloud.

- **Be curious and listen to understand.** Conversation is as much about listening as it is about talking. You might enjoy exploring how others' experiences have shaped their values and perspectives.
- **Show respect and suspend judgment.** People tend to judge one another. Setting judgement aside opens you up to learning from others and makes them feel respected and appreciated. Try to truly listen, without interruption or crosstalk.
- **Note any common ground as well as any differences.** Look for areas of agreement or shared values that may arise and take an interest in the differing beliefs and opinions of others.
- **Be authentic and welcome that from others.** Share what's important to you. Speak from your experience. Be considerate of others who are doing the same.
- **Be purposeful and to the point.** Do your best to keep your comments concise and relevant to the question you are answering. Be conscious of sharing airtime with other participants.
- **Own and guide the conversation.** Take responsibility for the quality of your participation and the conversation as a whole. Be proactive in getting yourself and others back on track if needed. Use an agreed upon signal like the "time out" sign if you feel the agreements are not being honored.

Question Rounds: What We'll Talk About

Optional: a participant can keep track of time and gently let people know when their time has elapsed.

Round One: Getting to Know Each Other (~10 min)

Each participant can take 1-2 minutes to answer one of these questions:

- What have you been thinking about since the last conversation?
- Have you done any deeper exploration into the topic since the previous conversation?

- Has the choice to participate in these discussions brought up any new questions for you as it relates to the topic(s)?

Round Two: Exploring the Topic -- Race and Faith Communities (~40 min)

One participant can volunteer to read this paragraph.

Race and religion have a long and complicated history, The Church of Jesus Christ of Latter-day Saints is no exception. The Church currently has a good relationship with the NAACP and all worthy members are able to fully participate in ordinances, but for much of its history the Church did not ordain men of black African descent. Our Religion or Spiritual path provides us a moral and practical foundation for our personal lives and communities. The uprisings for racial justice of the past years invite us to take a deeper look at our faith and the universal tenet of treating others as we would want to be treated. This conversation will help us explore the ways both overt and subtle racism may be present in our personal lives, on campus, and in our faith communities.

Definitions useful for this discussion:

- *anti-Blackness:* specific racial prejudice that devalues and marginalizes Black people
- *racism:* when patterns of policy, institutions, dominant ideologies and popular representations perpetuate social, political, and economic inequities between races
- *spiritual bypassing:* using spiritual ideas or practices to side-step complicated issues like race

Take ~2 minutes each to answer a question below without interruption or crosstalk. After everyone has answered, the group may take a few minutes for clarifying or follow up questions/responses. Continue exploring additional questions as time allows.

- What messages have you internalized from your faith's culture, history, and doctrine around Blackness, People of Color, and Whiteness?
- How has your faith inspired you to respond to recent events following the death of George Floyd and others? What are you feeling called to do? Where do you feel resistance?
- Have you seen or heard about instances of racism or "othering" on campus and/or your faith community? What challenges/barriers do you face in addressing them or speaking up?
- What can you do to support students of color on campus? What could the university do?
- What hopes and concerns do you have around anti-racism efforts on campus right now?

Round Three: Reflecting on the Conversation (~15 min)

Take 2 minutes to answer one of the following questions:

- What was most meaningful / valuable to you in this Living Room Conversation?
- What learning, new understanding or common ground was found on the topic?
- How has this conversation changed your perception of anyone in this group, including yourself?
- Is there a next step you would like to take based upon the conversation you just had?

Closing (~5 min)

- *Give us feedback!* Use livingroomconversations.org/feedback-form/ or QR code
- *Donate!* Make more of these possible; give at livingroomconversations.org/donate/
- *Join or host more conversations!* With a) this group by exchanging your emails; b) others in person and/or by video call online. Get more involved or learn how to host at livingroomconversations.org/get-involved/

This is an open source project. Please use, share and modify with attribution to LivingRoomConversations.org
